# Diabetes knowledge and glycemic control among type 2 diabetes patients at public hospitals in Debre Berhan, Ethiopia

**DOI:** 10.1371/journal.pone.0317288

**Published:** 2025-01-30

**Authors:** Girma Deshimo Lema, Enguday Demeke Gebeyaw

**Affiliations:** 1 Department of Internal Medicine, School of Medicine, Debre Berhan University, Debre Berhan, Ethiopia; 2 School of Public Health, Debre Berhan University, Debre Berhan, Ethiopia; Zagazig University Faculty of Human Medicine, EGYPT

## Abstract

**Background:**

Diabetes mellitus is a growing global health issue, especially in low- and middle-income countries like Ethiopia. To the best of our knowledge, the impact of diabetes knowledge on glycemic control in Ethiopia has not been documented. This study assessed diabetes knowledge and its relationship with glycemic control among Type 2 diabetes (T2DM) patients in Debre Berhan, Ethiopia.

**Methods:**

A cross-sectional study was conducted involving 380 patients diagnosed with T2DM who were receiving care at two hospitals in Debre Berhan from January 1 to March 30, 2024. Patients’ knowledge was assessed using the modified Diabetes Knowledge Questionnaire (DKQ-18), categorizing outcomes as either good or poor. Glycemic control was evaluated using hemoglobin A1c (HbA1c) levels. Logistic regression analyses were conducted to identify predictors of poor diabetes knowledge. Correlation analysis was used to evaluate the relationship between knowledge and glycemic control.

**Results:**

Among the 380 participants, 75.2% were older than 45 years, and 51.3% were male. Overall, 62.4% of participants had poor knowledge of diabetes. Additionally, 72.6% had poor glycemic control, with HbA1C levels ≥7%. The mean average diabetes knowledge score was 7.9 (SD = 3.49) out of 18, and the median HbA1C was 8%. Diabetes knowledge was significantly associated (*p* < 0.05) with patients’ educational level, occupation, family history of diabetes, and glycemic control. The Spearman correlation coefficient between HbA1C and diabetes knowledge scores was -0.166 (*p* = 0.001), suggesting a weak but statistically significant inverse relationship between knowledge scores and HbA1C levels.

**Conclusions:**

The study found that the majority of patients had a low level of diabetes knowledge. Enhancing diabetes education and identifying additional factors that influence glycemic control are crucial for achieving optimal diabetes management in Ethiopia. Public health initiatives should prioritize enhancing diabetes knowledge through targeted educational programs and resources to support effective diabetes management and achieve optimal glycemic control.

## 1. Background

Diabetes is a serious, chronic illness that arises when the pancreas fails to produce enough insulin or when the body is unable to effectively use the insulin it produces [1]. Type 2 diabetes (T2DM) is the most common form, affecting over 90% of people with diabetes worldwide [2]. As of 2021, there were 529 million people globally living with diabetes, across all age groups, resulting in a global age-standardized prevalence rate of 6.1%. The burden of diabetes is increasing most rapidly in low- and middle-income countries [3]. The prevalence of diabetes in Ethiopia is significantly increasing, with an overall rate of 6.5% [4].

Diabetes mellitus is known to be a significant contributor to early mortality and disability due to a range of microvascular and macrovascular complications. Poor glycemic control in type 2 diabetes increases the risk of complications, whereas maintaining effective glycemic control helps lower the risk of these complications such as cardiovascular disease, neuropathy, nephropathy, and retinopathy [5, 6]. T2DM can be prevented through lifestyle modifications such as maintaining a healthy weight, engaging in regular physical activity, and following a balanced diet [7].

Despite the availability of a growing array of medications developed to manage diabetes, including newer classes of drugs with various mechanisms of action, achieving and maintaining optimal glycemic control continues to be a significant challenge for many patients. Factors contributing to this challenge include individual variability in response to medication, adherence to treatment regimens, lifestyle factors such as diet and exercise, and the need for continuous monitoring and adjustment of therapy. Therefore, while advancements in medication provide more options, a comprehensive approach that includes patient education, regular follow-up, and personalized treatment plans is essential for overcoming these challenges and achieving better long-term outcomes in diabetes management [8–10].

Diabetes education plays a crucial role in managing the disease effectively. By improving patients’ understanding of their condition and enhancing their self-care practices, diabetes education helps achieve optimal glycemic control. This, in turn, leads to a reduction in diabetes-related complications, improving overall health outcomes and quality of life for individuals with the condition. It has been emphasized that addressing external factors, particularly social determinants of diabetes, is crucial for achieving sustainable improvements in diabetes outcomes [11–15].

Previous studies have explored the level of diabetes knowledge among patients and its impact on glycemic control [16–20]. Studies have indicated that diabetes knowledge among patients is often low. These studies have highlighted that many individuals with diabetes lack a comprehensive understanding of the condition, including its management and potential complications [18, 20–26].

Involving patients in developing treatment plans enhances adherence. Therefore, having a basic knowledge of diabetes is crucial for patients to engage effectively in decision-making and to benefit from additional education. In Ethiopia, a number of studies have explored diabetes knowledge among diabetic patients [14, 22, 26, 27]. To the best of our knowledge, the association between diabetes knowledge and glycemic control has not been documented. By identifying gaps in patients’ understanding of their condition, healthcare providers can develop targeted educational interventions to enhance self-management practices. This, in turn, can lead to improved patient outcomes and inform the development of more effective healthcare practices and policies. Therefore, this study aimed to assess diabetes knowledge and its relationship with glycemic control among Type 2 diabetes patients in Debre Berhan, Ethiopia.

## 2. Methods

### 2.1. Ethics statement

The study adhered to the Declaration of Helsinki and was approved by the Institutional Review Board of Asrat Woldeyes Health Science Campus, Debre Berhan University, under reference number IRB-192. Written informed consent was obtained from all participants before the study commenced. The patient’s privacy and confidentiality were protected.

### 2.2. Study setting and design

A cross-sectional study was conducted from January 1 to March 30, 2024, within the diabetic care services of two government hospitals in Debre Berhan city. Located in the North Shewa zone of Northeast Ethiopia, Debre Berhan is 130 km from Addis Ababa. The city hosts two public hospitals, both of which have outpatient clinics for diabetes management; one of these hospitals is a teaching facility. These diabetic care clinics provide services to approximately 600 patients with type 2 diabetes every month.

### 2.3. Study participants, sampling and recruitment procedures

The study included all adult patients ≥ 18 years of age who had been diagnosed with type 2 diabetes mellitus and had received follow-up care for at least three months prior to recruitment. Excluded from the study were patients who had not started diabetes medications.

**Fig 1:**
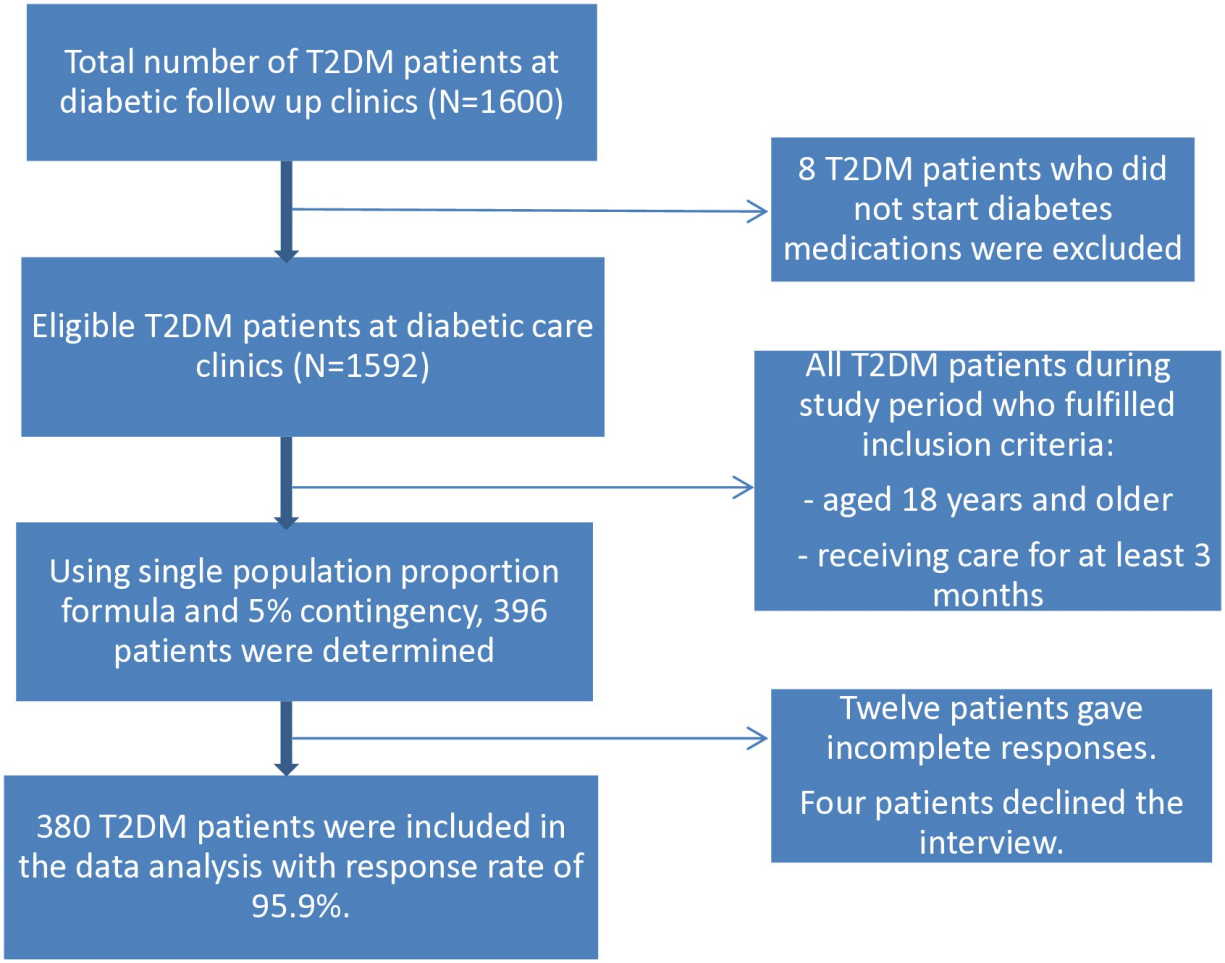
Summary of the study design and patient selection flowchart, 2024.

The sample size was determined using the single population proportion formula: N = Z^2^*p(1-p)/d^2^, where p = 56% [22] proportion (p), with a 95% confidence level (Z) and a margin of error (d) of 5%. This calculation resulted in a sample size of 378. After accounting for a 5% non-response rate, the required sample size was adjusted to 396 participants. Patients were selected using a simple random sampling technique during their visits for follow-up care or medication refills over a three-month period. To implement this technique, we first compiled a complete list of all attendees at the clinic during the study period, which served as our sampling frame. From this list, participants were randomly selected using a lottery method, ensuring that each patient had an equal chance of being included in the study. Fig 1 showed patient selection flowchart.

### 2.4. Data collection tools and procedures

An interviewer-guided self-administered questionnaire was initially prepared in English. It was then translated into the local language to ensure participants could understand it. To maintain accuracy and consistency, the questionnaire was subsequently back-translated into English. A pre-test of the questionnaire was conducted two weeks prior to the actual data collection, involving 5% of participants who were not part of the final analysis, to identify and address any issues or ambiguities. Data collectors received thorough training on using the data collection tool. Two trained health professionals carried out the data collection under the supervision of the principal investigator to ensure data quality. Completed questionnaires were collected and reviewed daily for completeness and consistency, with the principal investigator and data collectors verifying the accuracy and completeness of all responses. The questionnaire takes approximately 5 minutes to complete during data collection. It comprised three main sections: The first section addressed socio-demographic characteristics. The second section assessed clinical aspects related to diabetes mellitus, including HbA1c levels. The third section was the Diabetes Knowledge Questionnaire -18 [28], designed to assess patients’ knowledge about diabetes. This 18-item questionnaire was adopted for the study context. It evaluated diabetes-related knowledge in four key domains: diabetes etiology and symptoms, complications, diet and treatment, and nursing care.

### 2.5. Variables

The dependent variables were diabetes knowledge and glycemic control levels. Diabetes knowledge was assessed using the DKQ-18, which is a modified version of the DKQ-24. Scores were calculated based on correct answers, with points awarded for each correct response and no points given for incorrect answers. The total score for each subject was obtained by summing the points, with a maximum attainable score of 18 and a minimum of 0. Scores of ≥ 9 were categorized as good knowledge, while scores < 9 were categorized as poor knowledge. The internal consistency of the knowledge assessment tool was measured using Cronbach’s alpha, which was adequate at 0.78. Glycemic control was classified into two categories: poor or good. Poor glycemic control was defined as HbA1C level ≥ 7%, while good glycemic control was defined as HbA1C level less than 7% [29].

The independent variables included: socio-demographic and economic data, herbal medicine use, history of cigarette smoking, history of alcohol consumption, family history of diabetes, duration of diabetes, type of therapy, body mass index (BMI), and diabetes complication.

### 2.6. Data processing and analysis

SPSS version 25 software was used for analysis. Descriptive statistics such as frequency and percentage were computed for categorical variables. Continuous variables were presented as mean ± standard deviation or median. Normality tests, model fit assessments, and multicollinearity checks were performed. The robustness and reliability of the logistic regression model were evaluated using the Hosmer-Lemeshow goodness-of-fit test. To assess the association of poor diabetes knowledge with the outcome variables, bivariate and multi-variable logistic regression analysis was carried out. The Mann-Whitney test was used to determine differences in HbA1C levels between groups with poor and good diabetes knowledge. A correlation matrix was employed to evaluate the relationship between knowledge scores and glycemic control. Significant association was declared by odds ratio with 95% confidence interval at p-value < 0.05.

## 3. Results

### 3.1. Socio demographic and clinical characteristics

The study included 380 patients with type 2 diabetes mellitus who completed the survey, yielding a response rate of 95.9%. Of these participants, 51.3% were male, and the mean age was 55.15 ± 12.71 years. Most participants were permanent residents of urban areas (68.2%) and reported being married (62.4%). Additionally, 31.6% had a family history of diabetes. Among the respondents, 31.3% were over 60 years old, 28.6% were employed in government positions, and 20.3% were engaged in farming. A significant majority of patients (75.8%) were treated solely with oral hypoglycemic agents, with metformin being the most frequently prescribed medication (92%). Additionally, 46.8% of the patients had diabetes-related complications. The socio-demographic and clinical characteristics of study participants are summarized in Table 1.

**Table 1:** Socio-demographic and clinical characteristics of study participants, Debre Berhan, Ethiopia (N = 380), 2024.

Variable	Categories	Frequency(n)	Percent (%)
Sex	Male	195	51.3
	Female	185	48.7
Age	<30	8	2.1
	30–45	86	22.6
	46–60	167	43.9
	>60	119	31.3
Marital status	Single	66	17.4
	Married	237	62.4
	Divorced	27	7.1
	Widowed	50	13.2
Residence	Urban	259	68.2
	Rural	121	31.8
Educational status	Unable to write and read	93	24.5
	1–8 grade	96	25.3
	9–12 grade	66	17.4
	College and above	125	32.9
Average monthly income	<1500 ETB	17	4.5
	1500–2500 ETB	51	13.4
	>2500 ETB	312	82.1
Occupation	Government employed	107	28.2
	Private employed	71	18.7
	Housewife	57	15
	Farmer	77	20.3
	Retired	68	17.9
Duration of diabetes	<1 year	52	13.7
	1–5 years	199	52.4
	>5 years	129	33.9
Type of treatment	Insulin injection	47	12.4
	Oral hypoglycemic drugs*	288	75.8
	Insulin and oral tablets	45	11.8
Family history of diabetes	Yes	120	31.6
	No	260	68.4
History of alcohol drinking	Yes	174	45.8
	No	206	54.2
History of cigarrete smoking	Yes	5	1.3
	No	375	98.7
History of herbal medicine use	Yes	190	50
	No	190	50
BMI(kg/m^2^)	<18.5 (underweight)	22	5.8
	18.5–24.99(Normal)	183	48.2
	25–29.99 (overweight)	148	38.9
	>30 (Obese)	27	7.1
Diabetes related complications	Yes	178	46.8
	No	202	53.2
Comorbidities	Yes	252	66.3
	No	128	33.7
HbA1c level	< 7%	104	27.4
	≥ 7%	276	72.6

Abbreviation: ETB, Ethiopian Birr; BMI, Body mass index.

* Oral hypoglycemic drugs include: metformin, glibenclamide, and glimepiride

### 3.2. Diabetes knowledge and associated factors

The DKQ scores ranged from 0 to 18, with a mean score of 7.9 (SD = 3.49) and a median score of 8. Six patients answered all 18 questions incorrectly, while only two patients answered 17 out of 18 knowledge scale questions correctly. Out of 380 participants, 237 (62.4%) had poor knowledge levels, while 143 (37.6%) had good knowledge levels.

In multivariate logistic regression analysis, poor diabetes knowledge was significantly associated (p < 0.05) with illiteracy, working in a farming occupation, the absence of a family history of diabetes, and higher HbA1C level. Table 2 summarizes the factors independently linked to poor diabetes knowledge.

**Table 2:** Factors associated with level of knowledge on diabetes, Debre Berhan, Ethiopia (N = 380), 2024.

Variable	Categories	Level of diabetes knowledge (N = 380)		*p*-value	^a^AOR (95% CI)
		Poor, n (%)	Good, n (%)		
Sex	Male	116(59.5)	79(40.5)	0.969	0.98(0.58–1.69)
	Female	121(65.4)	64(34.6)		1
Age	<30	4(50)	4(50)		1
	30–45	43(50)	43(50)	0.851	0.85 (0.16–4.50)
	46–60	101(60.5)	66(39.5)	0.796	1.25 (0.23–6.65)
	>60	89(74.8)	30(25.2)	0.456	1.92 (0.35–10.63)
Educational status	Unable to write and read	75(80.6)	18(19.4)	0.002*	3.23 (1.53–6.84)
	1–8 grade	63(65.6)	33(34.4)	0.192	1.57 (0.79–3.08)
	9–12 grade	38(57.6)	28(42.4)	0.199	1.57 (0.79–3.12)
	College and above	61(48.8)	64(51.2)		1
Residence	Urban	153(59.1)	106(40.9)	0.085	0.62(0.36–1.07)
	Rural	84(69.4)	37(30.6)		1
Occupation	Government employed	50(46.7)	57(53.3)		1
	Private employed	35(49.3)	36(50.7)	0.129	0.57 (0.28–1.18)
	Housewife	41(71.9)	16(28.1)	0.392	1.49 (0.59–3.76)
	Farmer	63(81.8)	14(18.2)	0.011*	2.8 (1.26–6.27)
	Retired	48(70.6)	20(29.4)	0.147	1.81 (0.81–4.04)
Family history of diabetes	Yes	67(55.8)	53(44.2)	0.032*	1
	No	170(65.4)	90(34.6)		1.77 (1.05–2.97)
Herbal medicine use	Yes	127(66.8)	63(33.2)	0.099	1.50(0.93–2.43)
	No	110(57.9)	80(42.1)		1
Presence of comorbidity	Yes	167(66.3)	85(33.7)	0.216	1.40(0.82–2.38)
	No	70(54.7)	58(45.3)		1
Glycemic control	<7%	58(55.8)	46(44.2)		1
	≥7%	179(64.9)	97(35.1)	0.018*	1.92 (1.12–3.30)

*Significant association (P < 0.05). Abbreviation: AOR, adjusted odd ratio; CI, Confidence Interval

^**a**^The model was adjusted for sex, age, educational status, residence, occupation, family history of diabetes, herbal medicine use, presence of comorbidity, and glycemic control

### 3.3. The relationship between diabetes knowledge and glycemic control

Among the participants, the mean HbA1C level was 8.13% (SD = 1.86), with a median of 8%, a minimum of 4.1%, and a maximum of 14%. Of the 380 participants, 276 (72.6%) had poor glycemic control, with HbA1C levels ≥ 7%. The Mann-Whitney test indicated significant differences in HbA1C between groups with poor and good diabetes knowledge scores (*p* = 0.02). The Spearman correlation coefficient between HbA1C and diabetes knowledge scores was -0.166 (p = 0.001), suggesting a weak inverse relationship between knowledge scores and HbA1C levels (Table 3).

**Table 3:** Correlation matrix for diabetes knowledge score and HbA1c.

	Diabetes knowledge score	HbA1C
Diabetes knowledge score	1	- 0.166**
HbA1C	- 0.166**	1

**Correlation is significant at the 0.01 level (2-tailed).

## 4. Discussion

The study revealed that the majority (62.4%) of participants had poor knowledge regarding causes, management, and complications of diabetes. This finding was consistent with several other studies that have reported generally low levels of diabetes knowledge among patients [18, 20, 22, 25, 30, 31]. However, contrasting our findings, some studies report higher levels of diabetes knowledge [17, 32, 33]. This difference could be attributed to factors such as geographic location, socioeconomic status, and access to healthcare, which may influence diabetes education levels, as well as variations in the tools used for assessment.

The observation that previous studies conducted in Ethiopia also reported low levels of diabetes knowledge supports the findings of our study [14, 22]. The consistent reports of inadequate diabetes knowledge across these studies highlight a widespread issue, underscoring the need for targeted interventions to address educational gaps and enhance diabetes knowledge. This situation may reflect broader systemic challenges in diabetes education and healthcare delivery in Ethiopia, such as limited resources, insufficient training for healthcare providers and inadequate public health initiatives. Additionally, cultural attitudes and socioeconomic factors may contribute to the low levels of diabetes knowledge. Considering the low level of knowledge, it is crucial to develop and implement more effective and comprehensive educational programs.

In multivariate logistic regression analysis, poor diabetes knowledge was significantly associated with illiteracy, working in a farming occupation, the absence of a family history of diabetes, and higher HbA1C level. This is in accord with previous studies [11, 14, 17, 18, 22, 34, 35].

The significant association between illiteracy and poor diabetes knowledge underscores the crucial role of literacy in accessing and understanding health information. Individuals with lower literacy levels may face challenges in reading and comprehending educational materials, which can hinder their ability to manage diabetes effectively. This highlights the need for educational resources that are accessible to those with varying levels of literacy, such as visual aids or oral instruction.

Working in a farming occupation was associated with poor diabetes knowledge, which might be related to factors such as limited access to healthcare and educational resources, or long working hours that reduce opportunities for health education. Additionally, rural areas where farming is common might have fewer healthcare facilities or less access to diabetes education programs. Tailoring interventions to address the specific needs of individuals in farming communities could help bridge this gap.

Otherwise, age, gender, income, duration of diabetes, type of diabetes treatment, herbal medicine use, and diabetes complications did not show a significant association with the poor level of diabetes knowledge in the study participants in the multivariable analysis.

The current study found a significant difference in HbA1C levels between groups with poor and good diabetes knowledge scores consistent with previous studies [11, 18–20]. This suggests that individuals with better diabetes knowledge generally have more favorable HbA1C levels compared to those with lower knowledge. Additionally, the present study showed that as diabetes knowledge scores increase, HbA1C levels tend to decrease, although the relationship was weak. This suggests that while diabetes education is important, other factors also significantly influence effective diabetes management. Comprehensive diabetes management involves not only knowledge but also adherence to treatment, lifestyle changes, and regular monitoring.

### 4.1. Limitation of the study

This study had several limitations to consider. Its cross-sectional design limits the ability to establish causal relationships. Moreover, the data were restricted to patients with type 2 diabetes attending two public hospitals in Debre Berhan city, and therefore may not represent the broader population of individuals with diabetes in Ethiopia. The study also did not account for all potential confounding factors that could affect glycemic control.

### 4.2. Conclusion

The current study assessed diabetes knowledge and associated factors, as well as the relationship between disease knowledge and glycemic control. The study revealed that most patients had low level of diabetes knowledge, with illiteracy, absence of a family history of diabetes, higher hemoglobin A1C levels, and employment in farming being significant predictors of poor knowledge. Additionally, our study found a weak inverse relationship between diabetes knowledge scores and HbA1c levels, suggesting that higher knowledge was somewhat associated with better blood glucose management, though the association was not strong. These findings underscore the essential role of comprehensive diabetes education in enhancing glycemic control for individuals with Type 2 diabetes. To address these issues, public health initiatives should prioritize the development and implementation of targeted educational programs and resources aimed at improving diabetes knowledge. Such efforts are crucial for supporting effective diabetes management and achieving better health outcomes.

## Supporting information

S1 File Questioner used to assess diabetes knowledge and glycemic control among Type 2 diabetes patients, 2024. (DOCX)

S1 Data Data collected from Type 2 DM patients to assess diabetes knowledge and glycemic control, 2024. (DOC)

## Abbreviations

AOR: Adjusted odds ratio
BMI: Body mass index
CI: Confidence interval
DKQ: Diabetes Knowledge Questionnaire
HbA1c: Hemoglobin A1c
SD: Standard deviation
SPSS: Statistical package for the social sciences
T2DM: Type 2 diabetes mellitus
