# Supplementary material for: Diabetes knowledge and glycemic control among type 2 diabetes patients at public hospitals in Debre Berhan, Ethiopia
Source: PLoS One. 2025 Jan 30;20(1):e0317288. doi: 10.1371/journal.pone.0317288 (PMC11781714; doi:10.1371/journal.pone.0317288)
Supplement: S1 File — (DOCX) [file pone.0317288.s001.docx]

**Part I- Socio-demographic characteristics**

| S.no | Questions | Response | Remarks |
| --- | --- | --- | --- |
| Q1 | Sex of the patient | 1.Male □ 2.Female □ |  |
| Q2 | Age in years | ……years |  |
| Q3 | Residence | 1.Urban □ 2.Rural □ |  |
| Q4 | Religion? | 1. Orthodox □ 2, Protestant □ 3.Muslim □ 4. Others (specify) ---- |  |
| Q5 | Marital status? | 1. Single □ 2. Married □ 3. Divorced □ 4. Widowed □ |  |
| Q6 | Family income per month? | ………..Birr |  |
| Q7 | Occupation? | 1. Government employed □ 2. Private employed □ 3. Housewife □ 4.Farmer □ 5.Retired □ |  |
| Q8 | Educational status | 1. Unable to read and write □ 2. 1–8 Grade □ 3. 9–12 Grade □ 4. College and above □ |  |

**Part II; clinical and behavioral characteristics**

| S.no | Questions | Response | Remarks |
| --- | --- | --- | --- |
| Q1 | Type of diabetes | **1.** Type 1 □ 2. Type 2□ |  |
| Q2 | Duration of DM in years | ……..years |  |
| Q3 | Current medication | 1. Insulin injection□ 2. OHA□ 3. Insulin and OHA □ 4.deit only□ |  |
| Q4 | Family history of DM? | 1. Yes□ 2. No □ |  |
| Q5 | Have_you_ever_used_any_herbal medicine for the control of DM? | 1. Yes□ 2. No □ |  |
| Q6 | Current HgA1c | **….** |  |
| Q7 | If yes for Q5, what was that herbal drug? | 1. Moringa □ 2.Shiferaw□ 3.Tseenaadam □ 4.tosegn□ 5.habesh□ 6.Damakese□ 7.dimbilal□ 8.Armagusa□ 9. Rouz□ 10.Ret□ 11.Yewef medanit□ 12.feto□ 13.sama□ 14.if others specify….. |  |
| Q8 | For how long did you use herbal drugs | ……. |  |
| Q9 | Did you stopped pharm drugs during using herbal drugs | 1. Yes 2. No 3.I used before pharm drugs started |  |
| Q10 | What happen after taking herbal medicine for your symptom? | 1. It resolves 2. It persists 3.it aggravates 4. If other symptoms developed after herbal medicine use list….. |  |
| Q11 | Do you have DM related complications (review from card) | 1.yes □ 2.No □ |  |
| Q12 | Type of complication you have | 1. Peripheral neuropathy □ 2. DM retinopathy □ 3. Peripheral artery disease □ 4. DM nephropathy □ 5. Glaucoma □ 6. Cataract □ 7. If others, specify…… |  |
| Q13 | Do you forget to take your DM medication? | 1. Always □ 2. Usually □ 3. Sometimes □ 4. Rarely □ 5. Never □ |  |
| Q14 | Do you stop taking your DM medication for a while? | 1. Always □ 2. Usually □ 3. Sometimes □ 4. Rarely □ 5.Never □ |  |
| Q15 | Do you decide to skip one of your DM medications? | 1. Always □ 2. Usually □ 3. Sometimes □ 4. Rarely □ 5. Never □ |  |
| Q16 | Do you use your DM medication less than prescribed? | 1. Always □ 2. Usually □ 3. Sometimes □ 4. Rarely□ 5. Never □ |  |
|  | Do you change the dosage of your stroke medication? | 1. Always □ 2. Usually □ 3. Sometimes □ 4. Rarely□ 5. Never □ |  |
| Q17 | Do you have Co-morbidity | 1.yes □ 2.No □ |  |
| Q18 | Type of co-morbidity | **1.** HTN □ 2.DM □ 3. DM and HTN □ 4. Cardiac & HTN □ 5. Dyslipidemia □ 6. If other specify… |  |
| Q19 | Current HgA1c | **….** |  |
| Q20 | Body mass index | ……kg/m^2^ |  |
| Q21 | Did you smoke cigarrate for the previous 6 months | 1.yes □ 2.No □ | **If no,q23** |
| Q22 | If yes forQ21, how many packages you smoke per day | ------ |  |
| Q23 | Did you drink alcohol for the previous one year | 1. Yes □ 2. No □ |  |
| Q24 | If yes for Q23,what type of alcohol you drink” | 1. Tela □ 2. Catecala □ 3. Berr □ 4. draft □ 5.woine□ 6 if others, specify------- |  |
| Q25 | How much you drink per day | …….. |  |
| Q26 | How money times per week you drink | ……. |  |

**Part III; Diabetes knowledge questioner**

| S.no | Questions | Response | Remarks |
| --- | --- | --- | --- |
| **Q1** | Diabetes is caused by failure of the kidneys to keep sugar out of the urine | **1.** Yes □ 2. No □ 3. I don’t know □ |  |
| **Q2** | Kidneys produce insulin | **1.** Yes □ 2. No □ 3. I don’t know □ |  |
| **Q3** | In untreated diabetes, the amount of sugar in the blood usually increases | **1.** Yes □ 2. No □ 3. I don’t know □ |  |
| **Q4** | A fasting blood sugar level of 210 is too high | **1.** Yes □ 2. No □ 3. I don’t know □ |  |
| **Q5** | The best way to check my diabetes is by testing my urine. | **1.** Yes □ 2. No □ 3. I don’t know □ |  |
| **Q6** | An insulin reaction is caused by too much food | **1.** Yes □ 2. No □ 3. I don’t know □ |  |
| **Q7** | Medication is more important than diet and exercise to control my diabetes. | **1.** Yes □ 2. No □ 3. I don’t know □ |  |
| **Q8** | Diabetes often causes poor circulation. | **1.** Yes □ 2. No □ 3. I don’t know □ |  |
| **Q9** | Cuts and abrasions on diabetics heal more slowly | **1.** Yes □ 2. No □ 3. I don’t know □ |  |
| **Q10** | Diabetics should take extra care when cutting their toenails. | **1.** Yes □ 2. No □ 3. I don’t know □ |  |
| **Q11** | A person with diabetes should cleanse a cut with iodine and alcohol. | **1.** Yes □ 2. No □ 3. I don’t know □ |  |
| **Q12** | The way I prepare my food is as important as the foods I eat. | **1.** Yes □ 2. No □ 3. I don’t know □ |  |
| **Q13** | Diabetes can damage my kidneys | **1.** Yes □ 2. No □ 3. I don’t know □ |  |
| **Q14** | Diabetes can cause loss of feeling in my hands, fingers, and feet | **1.** Yes □ 2. No □ 3. I don’t know □ |  |
| **Q15** | Shaking and sweating are signs of high blood sugar. | **1.** Yes □ 2. No □ 3. I don’t know □ |  |
| **Q16** | Frequent urination and thirst are signs of low blood sugar | **1.** Yes □ 2. No □ 3. I don’t know □ |  |
| **Q17** | Tight elastic hose or socks are not bad for diabetics. | **1.** Yes □ 2. No □ 3. I don’t know □ |  |
| **Q18** | A diabetic diet consists mostly of special foods | **1.** Yes □ 2. No □ 3. I don’t know □ |  |
