# Supplementary material for: Diabetes knowledge and glycemic control among type 2 diabetes patients at public hospitals in Debre Berhan, Ethiopia
Source: PLoS One. 2025 Jan 30;20(1):e0317288. doi: 10.1371/journal.pone.0317288 (PMC11781714; doi:10.1371/journal.pone.0317288)
Supplement: S1 Data — (DOC) [file pone.0317288.s002.doc]

FREQUENCIES VARIABLES=Q1 VAR00109 Q5 Q3 Q8 Q7 VAR00120 VAR00135 Q11 Q12 Q26 VAR00042 VAR00044 Q19
    Q14 VAR00133
  /ORDER=ANALYSIS.


Frequencies


Notes	
Output Created	23-DEC-2024 05:36:55	
Comments		
Input	Data	C:\Users\user\Desktop\Accadamical\Internal medicine\Research methodology\2016\DM\Knowledge\main\Plos one\type 2 DMK data file.sav	
	Active Dataset	DataSet1	
	Filter	<none>	
	Weight	<none>	
	Split File	<none>	
	N of Rows in Working Data File	380	
Missing Value Handling	Definition of Missing	User-defined missing values are treated as missing.	
	Cases Used	Statistics are based on all cases with valid data.	
Syntax	FREQUENCIES VARIABLES=Q1 VAR00109 Q5 Q3 Q8 Q7 VAR00120 VAR00135 Q11 Q12 Q26 VAR00042 VAR00044 Q19
    Q14 VAR00133
  /ORDER=ANALYSIS.	
Resources	Processor Time	00:00:00.02	
	Elapsed Time	00:00:00.01	


Statistics	
	Sex of the patient	age categorised	Marital status of the patient	Residence of the patient	Educational status of the patient	
N	Valid	380	380	380	380	380	
	Missing	0	0	0	0	0	

Statistics	
	Occupation of the patient	income categorised	Duration of DM catagorized	Current medication	Family history of DM	
N	Valid	380	380	380	380	380	
	Missing	0	0	0	0	0	

Statistics	
	Do you have Co-morbidity	Did you smoke cigarrate for the previous 6 months	Did you drink alcohol for the previous one year	Do you have DM related complications (review from card)	History of herbal medicine use for the control of DM	
N	Valid	380	380	380	380	380	
	Missing	0	0	0	0	0	

Statistics	
	hemoglobin A1Ccat	
N	Valid	380	
	Missing	0	


Frequency Table


Sex of the patient	
	Frequency	Percent	Valid Percent	Cumulative Percent	
Valid	Male	195	51.3	51.3	51.3	
	Female	185	48.7	48.7	100.0	
	Total	380	100.0	100.0		


age categorised	
	Frequency	Percent	Valid Percent	Cumulative Percent	
Valid	<30	8	2.1	2.1	2.1	
	30-60	253	66.6	66.6	68.7	
	>60	119	31.3	31.3	100.0	
	Total	380	100.0	100.0		


Marital status of the patient	
	Frequency	Percent	Valid Percent	Cumulative Percent	
Valid	Single	66	17.4	17.4	17.4	
	Married	237	62.4	62.4	79.7	
	Divorced	27	7.1	7.1	86.8	
	Widowed	50	13.2	13.2	100.0	
	Total	380	100.0	100.0		


Residence of the patient	
	Frequency	Percent	Valid Percent	Cumulative Percent	
Valid	Urban	259	68.2	68.2	68.2	
	Rural	121	31.8	31.8	100.0	
	Total	380	100.0	100.0		


Educational status of the patient	
	Frequency	Percent	Valid Percent	Cumulative Percent	
Valid	Unable to read and write	93	24.5	24.5	24.5	
	1-8 Grade	96	25.3	25.3	49.7	
	9-12 Grade	66	17.4	17.4	67.1	
	College and above	125	32.9	32.9	100.0	
	Total	380	100.0	100.0		


Occupation of the patient	
	Frequency	Percent	Valid Percent	Cumulative Percent	
Valid	Government employed	107	28.2	28.2	28.2	
	Private employed	71	18.7	18.7	46.8	
	Housewife	57	15.0	15.0	61.8	
	Farmer	77	20.3	20.3	82.1	
	Retired	68	17.9	17.9	100.0	
	Total	380	100.0	100.0		


income categorised	
	Frequency	Percent	Valid Percent	Cumulative Percent	
Valid	<1500	17	4.5	4.5	4.5	
	1500-2500	51	13.4	13.4	17.9	
	>2500	312	82.1	82.1	100.0	
	Total	380	100.0	100.0		


Duration of DM catagorized	
	Frequency	Percent	Valid Percent	Cumulative Percent	
Valid	<1 yrs	52	13.7	13.7	13.7	
	1-5 yrs	199	52.4	52.4	66.1	
	>5 yrs	129	33.9	33.9	100.0	
	Total	380	100.0	100.0		


Current medication	
	Frequency	Percent	Valid Percent	Cumulative Percent	
Valid	Insulin injection	47	12.4	12.4	12.4	
	OHA	288	75.8	75.8	88.2	
	Insulin and OHA	45	11.8	11.8	100.0	
	Total	380	100.0	100.0		


Family history of DM	
	Frequency	Percent	Valid Percent	Cumulative Percent	
Valid	Yes	120	31.6	31.6	31.6	
	No	260	68.4	68.4	100.0	
	Total	380	100.0	100.0		


Do you have Co-morbidity	
	Frequency	Percent	Valid Percent	Cumulative Percent	
Valid	yes	252	66.3	66.3	66.3	
	No	128	33.7	33.7	100.0	
	Total	380	100.0	100.0		


Did you smoke cigarrate for the previous 6 months	
	Frequency	Percent	Valid Percent	Cumulative Percent	
Valid	yes	5	1.3	1.3	1.3	
	No	375	98.7	98.7	100.0	
	Total	380	100.0	100.0		


Did you drink alcohol for the previous one year	
	Frequency	Percent	Valid Percent	Cumulative Percent	
Valid	yes	174	45.8	45.8	45.8	
	No	206	54.2	54.2	100.0	
	Total	380	100.0	100.0		


Do you have DM related complications (review from card)	
	Frequency	Percent	Valid Percent	Cumulative Percent	
Valid	yes	178	46.8	46.8	46.8	
	No	202	53.2	53.2	100.0	
	Total	380	100.0	100.0		


History of herbal medicine use for the control of DM	
	Frequency	Percent	Valid Percent	Cumulative Percent	
Valid	Yes	190	50.0	50.0	50.0	
	No	190	50.0	50.0	100.0	
	Total	380	100.0	100.0		


hemoglobin A1Ccat	
	Frequency	Percent	Valid Percent	Cumulative Percent	
Valid	<7	104	27.4	27.4	27.4	
	>=7	276	72.6	72.6	100.0	
	Total	380	100.0	100.0		

FREQUENCIES VARIABLES=VAR00128 Q13
  /STATISTICS=STDDEV MINIMUM MAXIMUM MEAN MEDIAN
  /ORDER=ANALYSIS.


Frequencies


Notes	
Output Created	23-DEC-2024 06:23:06	
Comments		
Input	Data	C:\Users\user\Desktop\Accadamical\Internal medicine\Research methodology\2016\DM\Knowledge\main\Plos one\type 2 DMK data file.sav	
	Active Dataset	DataSet1	
	Filter	<none>	
	Weight	<none>	
	Split File	<none>	
	N of Rows in Working Data File	380	
Missing Value Handling	Definition of Missing	User-defined missing values are treated as missing.	
	Cases Used	Statistics are based on all cases with valid data.	
Syntax	FREQUENCIES VARIABLES=VAR00128 Q13
  /STATISTICS=STDDEV MINIMUM MAXIMUM MEAN MEDIAN
  /ORDER=ANALYSIS.	
Resources	Processor Time	00:00:00.03	
	Elapsed Time	00:00:00.02	


Statistics	
	diabetic knowledge score	Current HgA1c	
N	Valid	380	380	
	Missing	0	0	
Mean	7.9263	8.132526	
Median	8.0000	8.000000	
Std. Deviation	3.48988	1.8602435	
Minimum	.00	4.1000	
Maximum	17.00	14.0000	
